# *In vitro *pharmacokinetics of anti-psoriatic fumaric acid esters

**DOI:** 10.1186/1471-2210-4-22

**Published:** 2004-10-12

**Authors:** Nicolle HR Litjens, Elisabeth van Strijen, Co van Gulpen, Herman Mattie, Jaap T van Dissel, H Bing Thio, Peter H Nibbering

**Affiliations:** 1 Department of Infectious Diseases, Leiden University Medical Center, Leiden, The Netherlands; 2 Department of Dermatology and Venereology, Erasmus Medical Center, Rotterdam, The Netherlands

## Abstract

**Background:**

Psoriasis is a chronic inflammatory skin disease that can be successfully treated with a mixture of fumaric acid esters (FAE) formulated as enteric-coated tablets for oral use. These tablets consist of dimethylfumarate (DMF) and salts of monoethylfumarate (MEF) and its main bioactive metabolite is monomethylfumarate (MMF). Little is known about the pharmacokinetics of these FAE. The aim of the present study was to investigate the hydrolysis of DMF to MMF and the stability of MMF, DMF and MEF at *in vitro *conditions representing different body compartments.

**Results:**

DMF is hydrolyzed to MMF in an alkaline environment (pH 8), but not in an acidic environment (pH 1). In these conditions MMF and MEF remained intact during the period of analysis (6 h). Interestingly, DMF was hardly hydrolyzed to MMF in a buffer of pH 7.4, but was rapidly hydrolyzed in human serum having the same pH. Moreover, in whole blood the half-life of DMF was dramatically reduced as compared to serum. The concentrations of MMF and MEF in serum and whole blood decreased with increasing time. These data indicate that the majority of the FAE in the circulation are metabolized by one or more types of blood cells. Additional experiments with purified blood cell fractions resuspended in phosphate buffered saline (pH 7.4) revealed that at concentrations present in whole blood monocytes/lymphocytes, but not granulocytes and erythrocytes, effectively hydrolyzed DMF to MMF. Furthermore, in agreement with the data obtained with the pure components of the tablet, the enteric-coated tablet remained intact at pH 1, but rapidly dissolved at pH 8.

**Conclusion:**

Together, these *in vitro *data indicate that hydrolysis of DMF to MMF rapidly occurs at pH 8, resembling that within the small intestines, but not at pH 1 resembling the pH in the stomach. At both pHs MMF and MEF remained intact. These data explain the observation that after oral FAE intake MMF and MEF, but not DMF, can be readily detected in the circulation of human healthy volunteers and psoriasis patients.

## Background

Psoriasis is a chronic inflammatory skin disease characterized by epidermal hyperplasia and infiltration of inflammatory cells into skin lesions. Anti-psoriatic therapies are mainly anti-inflammatory. Long-term use of many of these anti-psoriatic therapies is often hampered by serious adverse effects [1-5]. In this connection it is of interest that already in 1959, Schweckendiek introduced fumaric acid, an intermediate of the citric acid cycle, for the treatment of his psoriasis [6]. The main adverse effect of fumaric acid therapy, i.e. induction of gastric ulcers, was overcome by application of a mixture of fumaric acid esters (FAE) with great bioavailability [7]. This mixture, consisting of dimethylfumarate (DMF) and salts of monoethylfumarate (MEF), was formulated as enteric-coated tablets. This systemic therapy, successfully applied by several German [8,9] and Dutch [10,11] dermatologists, can be taken by patients for a long period due to the excellent safety profile [12]. Adverse effects that do occur are mostly mild and transient and include facial flushing and gastro-intestinal complaints. Pharmacokinetic data of FAE therapy are very limited and mainly based on personal communications [8,13]. For such a pharmacokinetic study, we first developed a highly sensitive method to determine concentrations of FAE in human blood (Litjens *et al*., manuscript submitted). In the present study, we investigated the hydrolysis of DMF to its most bioactive metabolite monomethylfumarate (MMF) and the stability of MMF, DMF and MEF in different environments representing various body compartments using this methodology.

## Results

### Stability of FAE and hydrolysis of DMF to MMF in buffers of various pH

**Figure 1:**
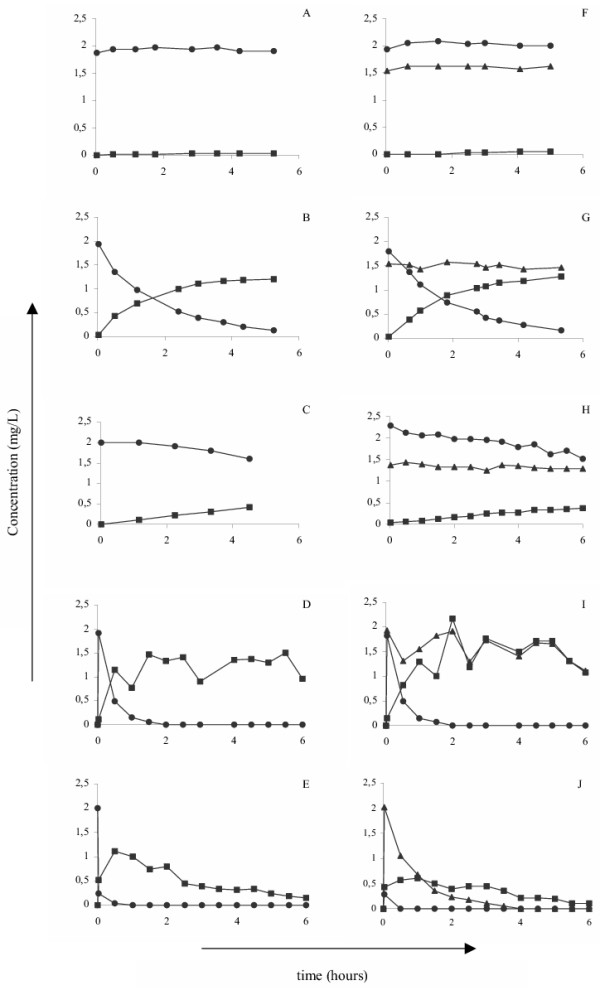
**Changes in the concentrations of the various FAE in different environments. **DMF at a concentration of 2 mg/L or the combination of 2 mg/L DMF and 1.4 mg/L MEF were placed at 37°C in 0.1 N HCl; pH 1 (A, F), 0.1 M sodium phosphate buffer; pH 8 (B, G), 0.1 M sodium phosphate buffer; pH 7.4 (C, H), normal human serum (D, I) or whole blood (E, J). At various intervals thereafter samples were collected and the MMF (squares), DMF (circles) and MEF (triangles) concentrations were measured using HPLC. Results are a representative experiment of at least 3 independent experiments.

DMF, MMF and MEF remained completely intact in a buffer of pH 1 mimicking the pH in the stomach (Figure 1A and 1F). However, at pH 8 resembling the pH in the small intestines DMF, the most abundant component of the FAE tablet, was hydrolyzed to MMF (the half-life of DMF amounted to 1.5 hr) (Figure 1B). Addition of MEF, the other component of the FAE tablet, did not affect the half-life of DMF (1.7 hr) (Figure 1G). MMF remained intact (Figure 1B) in this buffer during the period of analysis (6 hr) as did MEF (Figure 1G).

To further examine the pH-dependency of the hydrolysis of DMF to MMF, we measured concentrations of DMF and MMF in phosphate buffers (with pH ranging from 6.5–8) supplemented with DMF or the combination of DMF and MEF. The results revealed that the half-life of DMF dramatically decreased with increasing pH values and the maximal hydrolysis of DMF to MMF was seen at pH 8 (half life of DMF was 1.5 hr). For example, at pH 7.4 the half-life of DMF amounted to 12.7 ± 1.0 hr (n = 3) (Figure 1C). In agreement with these results we observed that the Fumaraat 120 tablet disintegrated completely between 1.5 and 2.5 hr in the alkaline, but not in the acidic, environment. The half-life of DMF in the tablet amounted to approximately 2.3 hr (data not shown).

### Changes in the concentrations of DMF, MMF and MEF in serum and whole blood

Since FAE must enter the circulation to exert their anti-psoriatic effects at the affected skin site [14], we determined the hydrolysis of DMF to MMF and examined the stability of MMF, DMF and MEF in both normal human serum and whole blood (both with a pH of 7.4). The half-life of DMF in serum (Table 1 and Figure 1D) is dramatically shorter (p < 0.05) than that in a buffer of the same pH. MMF (Figure 1D and 1I) and MEF (Figure 1I) concentrations slowly decreased in serum during the period of analysis (6 hr). Furthermore, the half-life of DMF was even shorter (p < 0.05) in whole blood than in serum (Table 1 and Figure 1E), indicating that circulating cells are also involved in the hydrolysis of DMF to MMF. Furthermore, concentrations of MMF (Figure 1E and 1J) and MEF (Figure 1J) in whole blood decreased steadily during the period of analysis (6 hr), indicating that they may be metabolized by blood cells as well.

**Table 1:** Hydrolysis rate of DMF to MMF and half-lives of DMF in different environments. To analyze under which circumstances DMF can be hydrolyzed to MMF and whether MEF affects the hydrolysis of DMF into MMF, we determined the hydrolysis rates for DMF in different environments. In short, a 0.1 M sodium phosphate buffer, human serum and whole blood (all pH 7.4) were spiked with either 2 mg/L of DMF or with the combination of 2 mg/L of DMF and 1.4 mg/L of MEF and at several intervals thereafter, samples were taken and prepared in order to measure the concentration of DMF, MMF and MEF by HPLC. Subsequently, after calculating the area under the curves for DMF (AUC_DMF) and MMF (AUC_MMF), the following model [16] was used to fit the concentrations of MMF and to estimate the k_DMF _(rate of hydrolysis of DMF into MMF) in these solutions: [MMF]_t = i _= (k_DMF_*AUC_DMF)-(k_MMF_*AUC_MMF) + [MMF]_t = 0. _In addition, the half-life was calculated using the following formula: t_1/2 _= ln(2)/k. Data are means and SD (n = 3). # and * significant (p < 0.05) different value between buffer and and serum and serum and whole blood, respectively.

	k_dmf _(h^-1^)	t_1/2 _(h)
**Buffer**		
Spiked with:		
DMF	0.06 (0.004)	12.72 (1.04)
DMF+MEF	0.05 (0.01)	15.17 (1.88)
**Serum**		
Spiked with:		
DMF	1.96 (0.47)	0.37 (0.08) #
DMF+MEF	2.20 (0.25)	0.32 (0.05) #
**Whole blood**		
Spiked with:		
DMF	8.01 (3.78)	0.10 (0.04)*
DMF+MEF	10.08 (2.74)	0.07 (0.02)*

**Figure 2:**
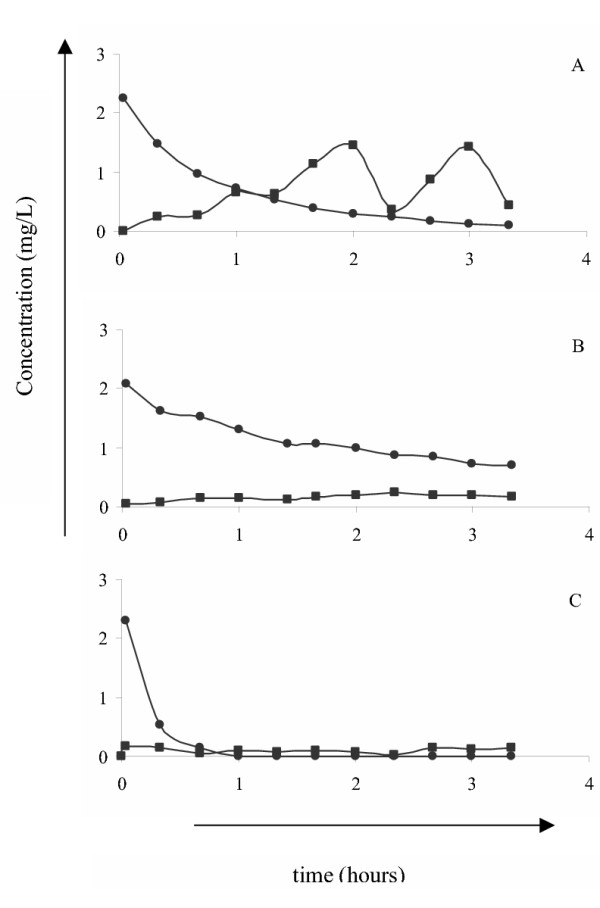
**Hydrolysis of DMF to MMF by various types of blood cells. **Monocytes/lymphocytes, granulocytes, and erythrocytes were purified from blood of healthy volunteers using centrifugational techniques. Next, the various cell types were resuspended in PBS pH 7.4 to concentrations present in whole blood, e.g. 1 × 10^6^/mL monocytes/lymphocytes (A), 5 × 10^6^/mL granulocytes (B) and 5 × 10^9^/mL erythrocytes (C), and then DMF was added to a final concentration of 2 mg/L. At various intervals thereafter samples were collected and the MMF (squares) and DMF (circles) concentrations were measured using HPLC. Results are a representative experiment of 3–4 independent experiments.

To find out which blood cell type(s) is (are) responsible for the hydrolysis of FAE in whole blood, hydrolysis of DMF in a buffer of pH 7.4 by purified blood cell fractions was analyzed. The results revealed that monocytes/lymphocytes (Figure 2A), but not granulocytes (Figure 2B) and erythrocytes (Figure 2C), at concentrations present in whole blood effectively hydrolyzed DMF to MMF.

## Discussion

A major finding of the present study is that hardly any DMF was hydrolyzed in a buffer of pH ≤ 7.4, whereas at pH 8, resembling the pH of the small intestines, this FAE was effectively hydrolyzed to its active metabolite MMF. It should be noted that MMF (and MEF) remained stable in these buffers. We realize that using acidic or alkaline buffers to mimick the conditions in body compartments, like the stomach and the small intestines, is only a first attempt to investigate the *in vitro *pharmacokinetics of FAE. For example, no enzymes, e.g. esterases, are present in these buffers whereas they are in these body compartments. In this connection, Werdenberg and collegues [15] recently showed that in the small intestines, the concentrations of MEF and MMF remained unaffected, whereas concentrations of DMF decreased by the action of esterases, such as carboxyl- and choline-esterases in this compartment. Esterase activity is also present in the liver which can cause a rapid disappearance of the various FAE from the circulation. Absorption of FAE from the small intestines into the circulation is not only dependent on the permeability of the intestinal membrane for the various FAE (permeability increases with increased acyl-chain length and increased lipophilicity), but also on the stabilities of the various FAE in the small intestines and liver. Clearly, hydrolysis of DMF to MMF is not only dependent on the pH of the environment but also on the activities of esterases.

Another important finding of this study is that the half-life of DMF in whole blood is considerably shorter than that in serum, although the pH of both blood and serum is 7.4. To explain this difference in hydrolysis of DMF in whole blood and serum we considered the possibility that blood cells also hydrolyze DMF to MMF. Using purified blood cell fractions resuspended in PBS (pH 7.4) we found that monocytes/lymphocytes, but not granulocytes and erythrocytes, at concentrations present in whole blood effectively hydrolyzed DMF to MMF. The rapid removal of DMF from PBS after addition of granulocytes and erythrocytes suggests that these blood cells bind DMF.

It should be realized that MMF (and MEF) most likely enter the circulation of psoriasis patients in order to exert their antipsoriatic effects in the skin lesions. In agreement we detected MMF and MEF, but not DMF, in the circulation of healthy volunteers and psoriasis patients after oral intake of Fumaraat 120^® ^tablets [[16], Litjens *et al*., manuscript submitted; Litjens *et al*., unpublished data]. Our observation that the MMF is more rapidly removed from whole blood than from serum could indicate that MMF (and MEF) is taken up by blood cells and perhaps further metabolized into FA, which subsequently fuels the citric acid cycle, as suggested earlier by Joshi (personal communication). The different interactions between FAE and blood cells may affect their functional activities, as has been reported earlier [14,17-19], thus contributing to the beneficial effects of FAE therapy.

## Conclusions

Together, these *in vitro *data indicate that DMF is almost completely hydrolyzed to MMF at an alkaline pH, but not at an acidic pH, suggesting that this hydrolysis occurs mainly within the small intestines and not in the stomach. Most likely, MMF and MEF are then absorbed in the circulation where they interact with blood cells and perhaps cells in the psoriatic lesions. The different interactions between these FAE and the various cell types may explain the beneficial effects of FAE in psoriasis. Finally, these *in vitro *experimental data will be key to the pharmacokinetic analysis of oral FAE in human healthy volunteers and psoriasis patients.

## Methods

### Fumaric acid esters (FAE)

The following FAE were used: dimethylfumarate (DMF; purity > 97%, TioFarma, Oud-Beijerland, The Netherlands), calcium-monoethylfumarate (MEF; purity > 97%, Tiofarma), monomethylfumarate (MMF; purity > 97%, AstraZeneca R&D, Charnwood, Loughborough, UK). In addition, the enteric-coated, magisterial manufactured tablet (named Fumaraat 120^®^; TioFarma), containing 120 mg of DMF and 95 mg of calcium-MEF was investigated in this study.

### DMF, MMF and MEF in acidic and alkaline environments

To investigate the stability of DMF, MMF and MEF and the hydrolysis of DMF to MMF in several environments representing various aspects of different body compartments, 0.1 N HCl with pH 1 and 0.1 M sodium phosphate buffer with pH 8 were spiked with 2 mg/L of DMF, MMF, MEF or the combination of 2 mg/L of DMF and 1.4 mg/L MEF, to resemble the ratio of these two components in the Fumaraat 120^® ^tablet. In addition, to determine the release of the contents of a Fumaraat 120^® ^tablet and the hydrolysis of DMF to MMF at pH 1 (0.1 N of HCl) and pH 8 (0.1 M of sodium phosphate buffer), the tablet was placed in these buffers and at various intervals samples were taken and prepared for measurement of the concentrations of DMF, MMF and MEF by high-performance liquid chromatography (HPLC) as described below.

To further investigate the effect of the pH on the hydrolysis of DMF to MMF, 0.1 M sodium phosphate buffers with pH values ranging from 6.5–8 were spiked with DMF and the combination of DMF and MEF. At several intervals thereafter, samples were taken, and then prepared for measurement of the various FAE by HPLC.

As the current buffers lack proteins, no extraction procedure was necessary and the concentrations of the various FAE in the samples could be directly quantified by HPLC (see below).

### Concentrations of DMF, MMF and MEF in serum and whole blood

As described above, serum and whole blood from 3 volunteers was spiked with 2 mg/L of DMF, MMF, MEF or the combination of 2 mg/L of DMF and 1.4 mg/L MEF. All volunteers were healthy as assessed by a full medical screening.

At several intervals, samples were taken, and then prepared for measurement of the various FAE by HPLC. In short, serum and whole blood contained proteins known to interfere with the measurement of FAE. To overcome this problem, the various FAE were extracted from serum and whole blood samples and subsequently the concentrations were measured by HPLC (see below).

### Effects of purified blood cell fractions on the hydrolysis of DMF in PBS (pH 7.4)

The various blood cell fractions were obtained from blood of healthy volunteers using centrifugational techniques as described earlier [17,19]. In short, blood was subjected to Ficoll Amidotrizoate (ρ = 1.077 gm/L; Dept. of Pharmacy, Leiden University Medical Center, Leiden, The Netherlands) density gradient centrifugation (440 g 20 min at 18°C). After resuspension of the cells in the pellet in phosphate buffered saline (PBS; pH 7,4) the granulocytes were purified by plasmasteril (Fresenius AG, Bad Homburg, Germany) sedimentation (1 g) for 10 min at 37°C, washed with PBS and the contaminating erythrocytes were lysed with distilled water. Erythrocytes were obtained after washing the cells in the Ficoll-Amidotrizoate pellet three times with PBS supplemented with 0.1 IU heparin. Cells in the Ficoll-Amidotrizoate interphase (monocytes/lymphocytes) were washed three times with PBS containing 0.5 IU heparin and then resuspended in PBS pH 7.4. Next, suspensions of 1 × 10^6 ^monocytes and lymphocytes/mL PBS, 4 × 10^6 ^granulocytes/mL PBS, and 5 × 10^9 ^erythrocytes/mL PBS were spiked with 2 mg/L DMF. Again, at several intervals samples were taken and concentrations of the various FAE were measured as described below.

### Sample preparation and HPLC analysis

The concentrations of the various fumarates in serum samples were determined as described (Litjens *et al*., submitted for publication). Briefly, after precipitation of serum proteins with acetonitrile, DMF in the samples was quantitated by HPLC. The sample preparation for MMF and MEF required a protein precipitation step with metaphosphoric acid followed by extraction with diethylether and additional pH-lowering to pH 0.5. Next, sodium chloride was added before centrifugation at 12,000 g. Thereafter, the ether layer was transferred to a glass vial and after evaporation the residue reconstituted in methanol: 0.1 M potassium phosphate buffer (KH_2_PO_4_/K_2_HPO_4_; pH 7.5) supplemented with 5 mM tetrabutylammonium dihydrogen phosphate 1:1 (v/v).

Concentrations of DMF, MMF, and MEF were determined on a HPLC apparatus (Spectra SERIES P100, Thermo Separation Products, Breda, The Netherlands) equipped with an Alltima C18 (5 μ 250*4.6; Alltech, Lokeren, Belgium) column and an Alltima Guard C18 precolumn (5 μ 7.5*4.6; Alltech, Lokeren, Belgium) using methanol:water 30:70 (v/v) as an eluent for DMF and methanol: potassium phosphate buffer supplemented with 5 mM tetrabutylammonium dihydrogen phosphate 20:80 (v/v) as eluent for MMF and MEF. The limit of detection for all three compounds amounted to 0.01 mg/L, the coefficient of variation for MMF, DMF and MEF was 7%, 8% and 9% at 0.5 mg/L, respectively (n = 4), and the recovery of MMF, DMF and MEF amounted to 75 ± 7%, 98 ± 3%, 67 ± 7% (n = 6). Standard curves constructed with purified FAE in buffers or human serum were used to quantify the concentrations of FAE in the various samples of these buffers and human serum or whole blood, respectively.

## Authors' contributions

NL participated in the design of the study, analysis of the data and drafted the manuscript. ES carried out the optimisation of the HPLC method and performed all HPLC analyses. CvG was responsible for the optimisation of the HPLC method and participated in the design of the study. HM participated in the design of the study and analysis of the data. JvD, HT and PN conceived of the study and participated in its design and coordination. All authors read and approved the final manuscript.
